# Facile distribution of an alkaline microenvironment improves human bone marrow mesenchymal stem cell osteogenesis on a titanium surface through the ITG/FAK/ALP pathway

**DOI:** 10.1186/s40729-021-00341-y

**Published:** 2021-06-28

**Authors:** Chen-Xi Wang, Ting Ma, Ming-Yue Wang, Hou-Zuo Guo, Xi-Yuan Ge, Yu Zhang, Ye Lin

**Affiliations:** 1 Department of Implantology, Peking University School and Hospital of Stomatology & National Clinical Research Center for Oral Diseases & National Engineering Laboratory for Digital and Material Technology of Stomatology & Beijing Key Laboratory of Digital Stomatology, Beijing, 100081 People’s Republic of China; 2 Central Laboratory, Peking University School and Hospital of Stomatology, Beijing, 100081 People’s Republic of China

**Keywords:** Dental implant, Titanium surface, Bone mesenchymal stem cells, Osteogenic differentiation, Microenvironment pH

## Abstract

**Purpose:**

Osseointegration at the titanium surface-bone interface is one of the key factors affecting the success rate of dental implants. However, the titanium surface always forms a passive oxide layer and impacts bone marrow–derived mesenchymal stem cell (BMSC) osteogenic differentiation after exposure to the atmosphere, which further leads to poor osseointegration. Given that wet storage helps prevent titanium aging and that weakly alkaline conditions stimulate BMSC osteogenic differentiation, the aim of the present study was to explore whether sodium bicarbonate, a well-known hydrogen ion (pH) buffer, forms an alkaline microenvironment on titanium surfaces to promote BMSC osteogenic differentiation.

**Material and methods:**

In this work, sand-blasted and acid-etched (SLA) titanium discs were soaked in 20 mM, 50 mM, 100 mM, and 200 mM sodium bicarbonate at room temperature for 5 min without rinsing. The influence of this surface modification on BMSC adhesion, proliferation, and osteogenic differentiation was measured. Additionally, cellular osteogenic differentiation–associated signaling pathways were evaluated.

**Results:**

We showed that titanium discs treated with sodium bicarbonate created an extracellular environment with a higher pH for BMSCs than the normal physiological value for 5 days, strongly promoting BMSC osteogenic differentiation via the activation of integrin-focal adhesion kinase-alkaline phosphatase (Itg-FAK-ALP). In addition, the proliferation and adhesion of BMSCs were increased after alkaline treatment. These cellular effects were most significant with 100 mM sodium bicarbonate.

**Conclusion:**

The results indicated that the titanium surface treated with sodium bicarbonate improved BMSC osteogenic differentiation mainly by creating an alkaline microenvironment, which further activated the Itg-FAK-ALP signaling pathway.

**Clinical relevance:**

Surfaces modified with 100 mM sodium bicarbonate had the highest initial pH value and thus showed the greatest potential to improve BMSC performance on titanium surfaces, identifying a novel conservation method for dental implants.

## Introduction

Dental implants have been widely used to treat patients with partial or complete edentulism and show long-term stability and a high survival rate [1, 2]. Titanium is currently the most extensively applied biomedical implant material due to its strong anticorrosive characteristics, biocompatibility, and mechanical resistance [3]. Although titanium is known to be a good osteoconductive material, previous research has shown that it always forms a passive and protective surface oxide layer once exposed to the atmosphere [4], which further leads to poor osteointegration between bone and implant interfaces.

Currently, most commercially used titanium implants are provided in gas-permeable packaging with a combination of micro- and nanoscale surface roughness modified by sandblasting and acid etching (SLA) [5, 6]. This topology has been reported to promote new bone formation and enhance osteointegration [7]. However, because hydrocarbons continually accumulate on fresh titanium surfaces, the zeta potential on the surface changes from positive to negative and blocks the Arg-Gly-Asp (RGD) sequence, a cell adhesion terminal structure, which further leads to time-dependent degradation of titanium osteoconductivity [8]. Several methods have been published to reactivate aged titanium surfaces, such as ultraviolet (UV) and nonthermal atmospheric pressure plasma (NTAPPJ) treatment [9, 10]. Studies have shown that the effect of UV treatment could be maintained for only a short period, which could barely meet the criteria of dental clinical conservation [10–13]. In addition, it would be difficult for dentists to apply UV treatment to a titanium surface immediately before use, and this method may result in an increased risk of infection.

Some authors reported that storing titanium implants in alendronate (ALN) or ddH_2_O could prevent the accumulation of organic impurities and maintain a hydrophilic surface [5, 14]. In addition, some authors believe that ALN can also directly stimulate osteoblast activity by increasing alkaline phosphatase (ALP) activity. However, ALN is mainly used as a bone antiresorptive agent by inhibiting osteoclastic activity, and its effect on osteoblasts remains unclear [15, 16]. Its clinical application is also limited by potential complications [17]. Our previous work showed that soaking in sodium bicarbonate, a mild alkaline compound with low biohazard potential, changed hydrophobic SLA titanium surfaces to hydrophilic and further improved osteoblast early adhesion and differentiation in vitro [18, 19]. Moreover, sodium bicarbonate is a well-known pH buffer that regulates acid and base equilibrium by hydrolysis and ionization reactions. This process is influenced by the concentration of sodium bicarbonate and environmental temperature. The extracellular pH (pH_e_) regulates the balance of resorption and formation/mineralization [20], and a more alkaloid environment could promote mineralization by increasing osteoblast activation [21]. Therefore, a proper concentration of sodium bicarbonate has the potential to avoid the aging and enhanced osseointegration of titanium implants. To the best of our knowledge, few studies have evaluated whether SLA titanium discs soaked in solutions with different pH values regulate seeded osteoblast fates.

In this study, we aimed to focus on the concentration of sodium bicarbonate used to soak SLA titanium discs. We investigated the characteristics of titanium surfaces after modification by sodium bicarbonate and evaluated the effect of varying concentrations of sodium bicarbonate on bone marrow mesenchymal stem cell (BMSC) adhesion, proliferation, and differentiation.

## Materials and methods

### Titanium specimen preparation and assessment

#### Titanium specimen preparation

Commercially pure titanium discs (ISO 5832-2 grade 4) with 1 mm thickness and 15 mm diameters were prepared as previously described [18, 19]. In brief, the SLA surface modification was obtained by blasting with grits of 0.25–0.50 m Al_2_O_3_ and acid etching followed by etching with hot HCl (10–16%)/H_2_SO_4_ (68–75%) at a temperature of 80–90 °C. All discs were then cleaned, rinsed, and dried with nitric acid, ddH_2_O, and air. Finally, the discs were stored in aluminum foil for further experiments (Wego Jericom Biomaterials Co., Weihai, China).

The alkaline treatment procedure was performed by immersing the discs in different concentrations of sodium bicarbonate (20 mM, 50 mM, 100 mM, and 200 mM) for 5 min at room temperature. All discs were then blow-dried in a nitrogen stream without rinsing before being used. The pH of each solution was measured by a pH meter (Mettler, Switzerland) at the same time.

#### Surface hydrophilicity assessment

The hydrophilicity of SLA titanium discs treated with different concentrations of sodium bicarbonate was examined both quantitatively and qualitatively by the water contact angle (WCA) test, which is performed with a 10-μL drop of ddH_2_O on the titanium surfaces. The measurement was performed by a contact angle system (OCA20, Dataphysics, Germany). All of the WCA data were obtained by ellipse methods. In this measurement process, the average WCAs of the 4 samples in each group were evaluated and then compared.

#### Surface microstructure characterization

For determination of the microstructure of SLA titanium discs treated with different concentrations of sodium bicarbonate, scanning electron microscopy (SEM) (S-3000N, Hitachi, Japan) and three-dimensional (3D) optical microscopy (3D-OPT) (Contour GT, Bruker, US) were applied to analyze the specimens before and after a 5-min sodium bicarbonate treatment. A total of 5 min could be a suitable treatment time according to previous research [18].

The SEM-scanned samples were coated with gold at 15.0 kV and examined at a magnification of × 2000. Surface roughness data and 3D images of the specimens were acquired by 3D-OPT. Roughness data were then analyzed with Vision64 software. Three specimens from each group were evaluated by observing ten random spots on each of them. The average roughness (*R*_a_) and surface roughness (*S*_a_) values were calculated.

### In vitro cytocompatibility and differentiation evaluation

#### BMSC culture

BMSCs (passage 2) were purchased from Cyagen Biosciences Technology (Cyagen, Guangzhou, China). The cells were cultured according to the manufacturer’s instructions. Briefly, BMSCs were cultured in α-minimal essential media (α-MEM, Gibco, USA) containing 10% fetal bovine serum (FBS, Gibco, USA) and 1% penicillin/streptomycin at 37 °C in a humidified atmosphere of 95% air and 5% CO_2_ with a culture media change every 2–3 days.

#### BMSC adhesion and spreading

For the spreading assay, BMSCs were plated at 5 × 10^4^ cells on each titanium surface treated with different concentrations of sodium bicarbonate. After 2 h, 4 h, and 24 h of incubation, the cells were washed with phosphate-buffered saline (PBS) and fixed with 4% paraformaldehyde for 30 min. Then, the cells were permeated with 0.1% Triton-X for 5 min, followed by staining with phalloidin-FITC (Sigma, USA) for 40 min at room temperature. After five washes with PBS, the nuclei were counterstained with DAPI (Solarbio, China) for 5 min. Sample images were captured with a fluorescence microscope.

For the SEM analyses, BMSCs were plated on titanium discs as described above, washed with PBS for 3 times, and fixed with 2% glutaraldehyde for 1 h. Then, the samples were subjected to sequential dehydration with a graded series of ethanol solutions (50%, 60%, 70%, 80%, 90%, 99%, and anhydrous ethanol) for 10 min each. Before the observation, all discs were dried in a freezing dryer overnight (Hitachi, Japan) and coated with platinum–palladium with an ion sputter machine (Hitachi, Japan).

#### BMSC proliferation

BMSCs were plated on SLA titanium discs treated with different concentrations of sodium bicarbonate at a density of 1 × 10^4^ cells/cm^2^ for 1, 3, 5, and 7 days. Cell proliferation was determined by a Cell Counting Kit-8 assay (CCK-8, Dojindo, Japan). CCK-8 was added and then incubated at 37 °C for 2 h. The optical density (OD) was recorded on an ELX-808 Absorbance Microplate Recorder (BioTek, Winooski, VT) at 450 nm.

#### Osteogenic differentiation of BMSCs

For osteogenic differentiation, BMSCs were seeded in six-well plates covered with prepared titanium discs at a density of 1 × 10^4^ cells/cm^2^. When the cells reached 80–90% confluence, the culture media was replaced with osteogenic media (Cyagen Biosciences, China) containing 10% FBS, 1% penicillin-streptomycin, 0.2 mM ascorbic acid, 10 mM β-glycerophosphate, and 0.1 μM dexamethasone. The media was changed 3–4 days. After 21 days, osteogenic differentiation was evaluated by Alizarin Red staining (Cyagen, Guangzhou, China). For the quantification analysis, the stained discs were desorbed using 10% cetylpyridinium chloride (Sigma, USA). Absorbance values at 590 nm were recorded. Total protein content was used for normalization and was determined by a protein concentration determination (BCA) assay kit (Thermo, USA).

#### In vitro cell culture media pH

BMSCs were cultured on SLA titanium discs treated with different concentrations of sodium bicarbonate following osteogenic differentiation. The culture media were collected at 1, 3, 5, and 7 days after cell seeding on the discs. The pH value of the cell culture media was further determined by a pH meter (Mettler, USA).

#### Alkaline phosphatase activity assay and ALP staining

For determination of ALP activity, BMSCs were seeded onto different SLA titanium discs at a density of 2 × 10^4^ cells per well. After a 7-day osteogenic differentiation procedure, the ALP activity was measured by an ALP activity kit (JianCheng Bioengineering Institute, Nanjing, China) according to the manufacturer’s instructions. The results were normalized to the levels of total protein, which were measured by the BCA method (Thermo, USA).

For alkaline phosphatase staining, after 7 days of osteogenic differentiation, the samples were washed with PBS solution three times at room temperature, and the cells were then fixed in 4% paraformaldehyde for 30 min and stained with a BCIP/NBT Alkaline Phosphatase Color Development kit (Beyotime Institute of Biotechnology, China) for 15 min. PBS was used for several washes, and the samples were analyzed by microscopy.

#### Quantitative real-time PCR

Total mRNA was isolated from the cells plated on different titanium surfaces by TRIzol reagent (Invitrogen, USA). The PrimeScript RT Reagent Kit (TaKaRa, Japan) was used to reverse-transcribe mRNA into cDNA, according to the manufacturer’s instructions. FastStart Universal SYBR Green Master Mix (ROX, USA) was mixed with cDNA, and qRT-PCR was performed on an ABI 7500 Real-Time PCR system (Applied Biosystems, USA). Relative quantification was performed with the △△Ct method, and normalization was performed with the housekeeping gene glyceraldehyde 3-phosphate dehydrogenase (GAPDH). The sequences of gene primers used for qRT-PCR are listed in Table 1.

**Table 1:** The primers for qRT-PCR

Genes	Forward sequences	Reverse sequences
GAPDH	5′-AACTTTGGCATTGTGGAAGG-3′	5′-ACACATTGGGGGTAGGAACA-3′
Itg α2	5′-TTAGCGCTCAGTCAAGGCAT-3′	5′-CGGTTCTCAGGAAAGCCACT-3′
Itg αV	5′-TTGTTTCAGGAGTTCCAAGA-3′	5′-TGAAGAGAGGTGCTCCAATA-3′
Itg β1	5′-GCAGGCGTGGTTGCCGGAAT-3′	5′-TTTTCACCCGTGTCCCACTTGGC-3′
Col1-α1	5′-CCAACGAGATCGAGCTCAGG-3′	5′-GACTGTCTTGCCCCAAGTTCC-3′
Runx2	5′-TCTTCCCAAAGCCAGAGCG-3′	5′-TGCCATTCGAGGTGGTCG-3′
ALP	5′-ACTCTCCGAGATGGTGGTGG-3′	5′- CGTGGTCAATTCTGCCTCCTT-3′
OPG	5′-TAACGTGATGAGCGTACGGG-3′	5′-GCAGCACAGCAACTTGTTCA-3′
RankL	5′-GAACACGCGTATTTACCTGC-3′	5′-CCCATAGCCCACATGCAGTT-3
TGF-β1	5′-CGACTCGCCAGAGTGGTTAT-3′	5′-AGTGAACCCGTTGATGTCCA-3′
BMP-2	5′-TCAAGCCAAACACAAACAGC-3′	5′-AGCCACAATCCAGTCATTCC-3′
OSX	5′-CCTCAGGCCACCCGTTG-3′	5′-AGCCATAGTGAACTTCCTCCTC-3′
OCN	5′-CAGTAAGGTGGTGAATAGAC-3′	5′-GGTGCCATAGATGCGCTTG-3′

#### Statistical analysis

Data were collected from three independent experiments and are expressed as the mean ± standard deviation (SD). One-way analysis (ANOVA) followed by Tukey’s test was performed by the GraphPad Prism 7 statistical software package (GraphPad Software, San Diego, USA) to analyze statistically significant differences (*P*) between the various groups. *P* < 0.05, *P* < 0.01, and *P* < 0.001 are represented symbolically by “*,” “**,” and “***,” respectively.

## Results

### Surface preparation and characterization

**Fig. 1:**
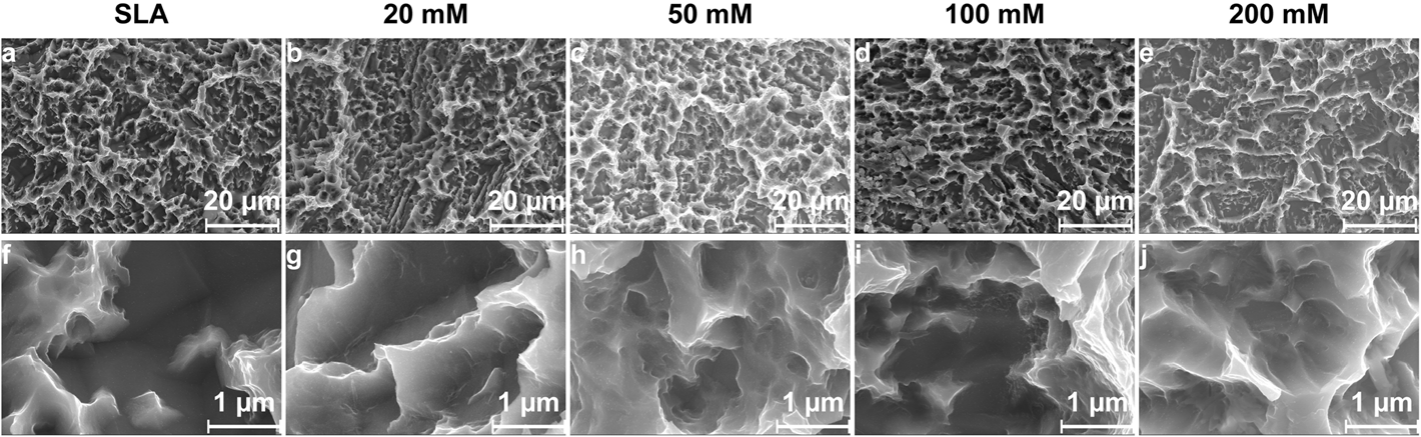
SEM images of surface morphology from SLA (**a**), 20 mM sodium bicarbonate@SLA (**b**), 50 mM sodium bicarbonate@SLA (**c**), 100 mM sodium bicarbonate@SLA (**d**), and 200 mM sodium bicarbonate@SLA discs (**e**) and a high magnification view of each group (**f**–**j**)

**Fig. 2:**
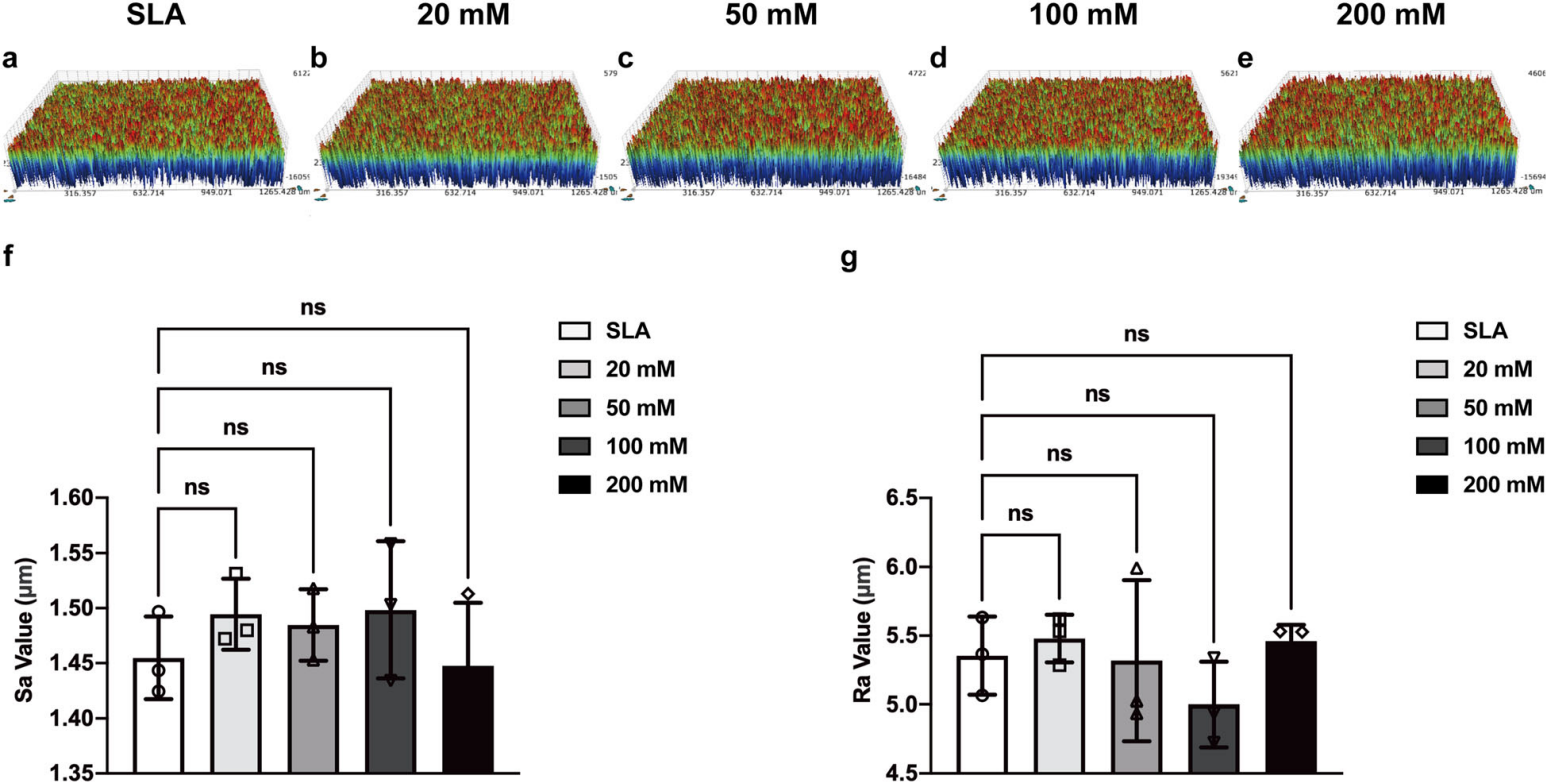
Three-dimensional optical microscopy images of the surface morphology from SLA (**a**), 20 mM sodium bicarbonate@SLA (**b**), 50 mM sodium bicarbonate@SLA (**c**), 100 mM sodium bicarbonate@SLA (**d**), and 200 mM sodium bicarbonate@SLA discs (**e**). Quantitative measurement of the roughness of each group by the *S*_a_ value (**f**) and the *R*_a_ value (**g**). Data are presented as the mean ± standard deviation (*n* = 3, *P< 0.05; **P < 0.01; ***P < 0.001)

**Fig. 3:**
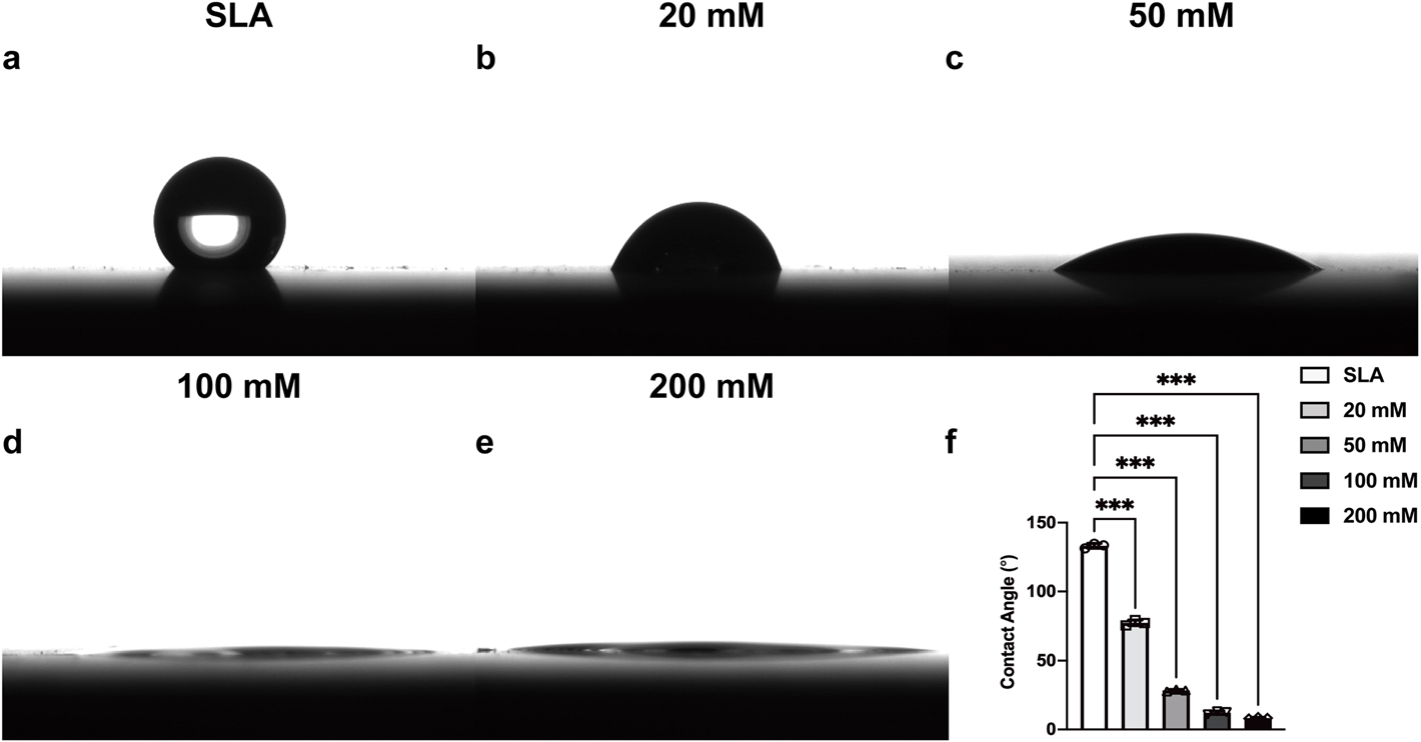
Water contact angle (WCA) images of a 10 휇L distilled water drop on SLA (**a**), 20 mM sodium bicarbonate@SLA (**b**), 50 mM sodium bicarbonate@SLA (**c**), 100 mM sodium bicarbonate@SLA (**d**), and 200 mM sodium bicarbonate@SLA discs (**e**). Quantitative results of the WCA of each group (**f**). Data are presented as the mean ± standard deviation (*n* = 3, *P < 0.05; **P < 0.01; ***P < 0.001).

**Fig. 4:**
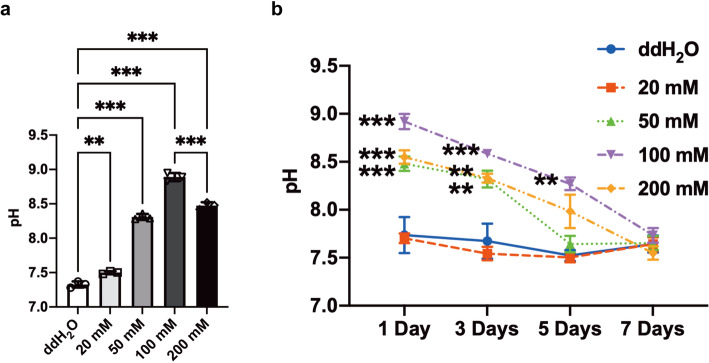
pH value of different concentrations (0 mM, 20 mM, 50 mM, 100 mM and 200 mM) of sodium bicarbonate solutions (**a**); variation of the pH value in culture media with time post cell seeding on SLA surfaces treated with different concentrations of sodium bicarbonate (**b**). Data are presented as the mean ± standard deviation (*n* = 3, *P < 0.05; **P < 0.01; ***P < 0.001)

This study used SLA- and sodium bicarbonate-treated titanium discs. The surface microtopographic characteristics measured by SEM and 3D-OPT were consistent with previously published data [18]. SEM showed that immersion in sodium bicarbonate at different concentrations had little influence on the microtopography of SLA titanium surfaces (Fig. 1). The 3D-OPT images further confirmed this result and displayed no difference between each group in surface roughness (Fig. 2). The WCA test demonstrated that after SLA treatment, titanium discs were hydrophobic (Fig. 3a); however, after immersion in different concentrations of sodium bicarbonate for 5 min, the surface became hydrophilic in a dose-dependent manner (Fig. 3b–f). In vitro measurements of the sodium bicarbonate pH used in each group showed that the pH of 100 mM sodium bicarbonate reached a maximum of 8.89 ± 0.12 (Fig. 4a).

### In vitro cell culture media pH

The pH of the cell culture media was determined by using a pH meter at 1, 3, 5, and 7 days after cells were seeded on the discs. There was a significant difference between all the sodium bicarbonate treatment groups and the control SLA group before osteogenesis. Sodium bicarbonate treatment effectively increased the cell culture medium pH. This effect diminished from day 4 except in the discs treated with 100 mM sodium bicarbonate until day 7 (Fig. 4b).

### BMSC adhesion and spreading

**Fig. 5:**
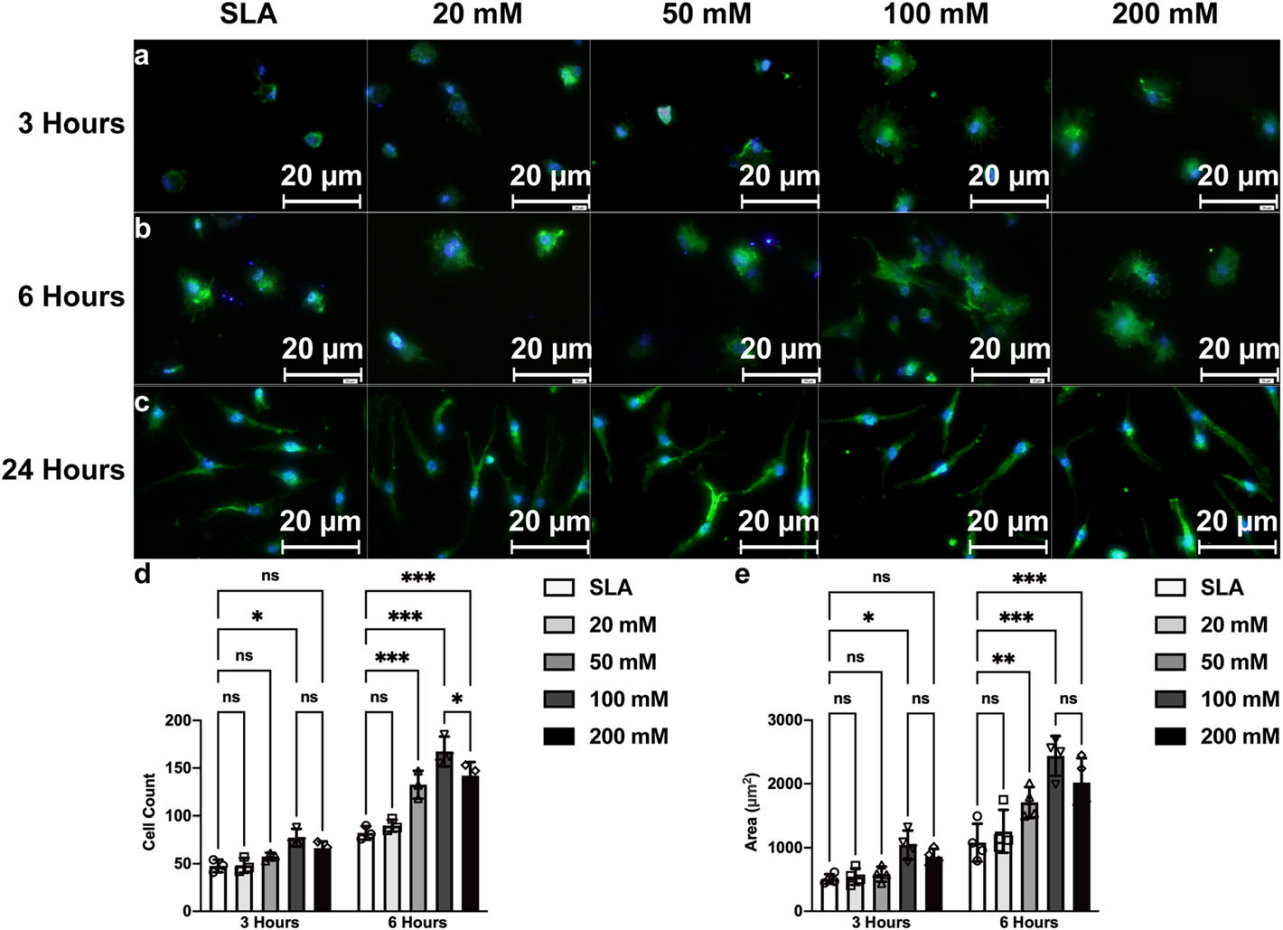
Fluorescence microscopy images of BMSCs cultured on SLA surfaces treated with different concentrations of sodium bicarbonate for 3 h (**a**), 6 h (**b**), and 24 h (**c**). Number of BMSCs in all groups at 3 and 6 h after cell seeding (**d**). The cellular area was measured with ImageJ for all groups at 3 and 6 h (**e**). Images were taken by selecting ten random areas, and data are presented as the mean ± standard deviation (*n* = 3, *P < 0.05; **P < 0.01; ***P < 0.001)

To evaluate BMSC adhesion and spreading on different SLA titanium surfaces, we counted the attached cell number and stretching areas with immunofluorescence (Fig. 5a–c). The results showed that SLA titanium discs treated with 100 mM sodium bicarbonate enhanced BMSC adhesion at 3 h post seeding. After 6 h, the SLA titanium discs treated with 50 mM, 100 mM, and 200 mM sodium bicarbonate exhibited increased cell adhesion (Fig. 5d). This result indicated that early cell adhesion on the SLA surfaces might be enhanced by proper alkaline treatments, which is consistent with our previous study. After 3 h of incubation, we also found that sodium bicarbonate had an effect on cell spreading. The BMSC spreading area with more filopodia processes was significantly greater on 100 mM sodium bicarbonate-treated discs than the other discs. SLA discs treated with 50 mM, 100 mM, and 200 mM sodium bicarbonate stimulated cell stretching (Fig. 5e). No significant difference was found at 24 h for either cell number or stretching in all groups, which is probably because the adhesion and spreading after 24 h already reached the maximum.

**Fig. 6:**
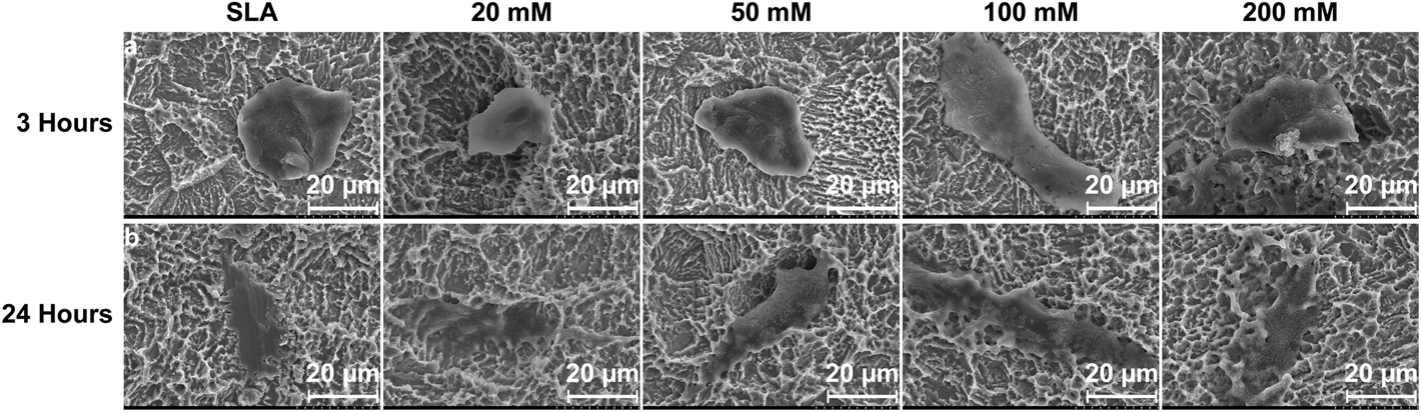
SEM images of BMSCs cultured on SLA surfaces treated with different concentrations of sodium bicarbonate for 3 h (**a**) and 24 h (**b**)

By SEM analysis, after a 3-h culture, BMSCs cells exhibited a flatter and more elongated morphology on the SLA treated with 100 mM sodium bicarbonate than other groups (Fig. 6a). No morphological differences were observed at 24 h, which is consistent with the immunofluorescence results (Fig. 6b). Taken together, 100 mM sodium bicarbonate appeared to be the most optical situation for BMSCs adhesion and spreading in our experiment.

### BMSC proliferation

**Fig. 7:**
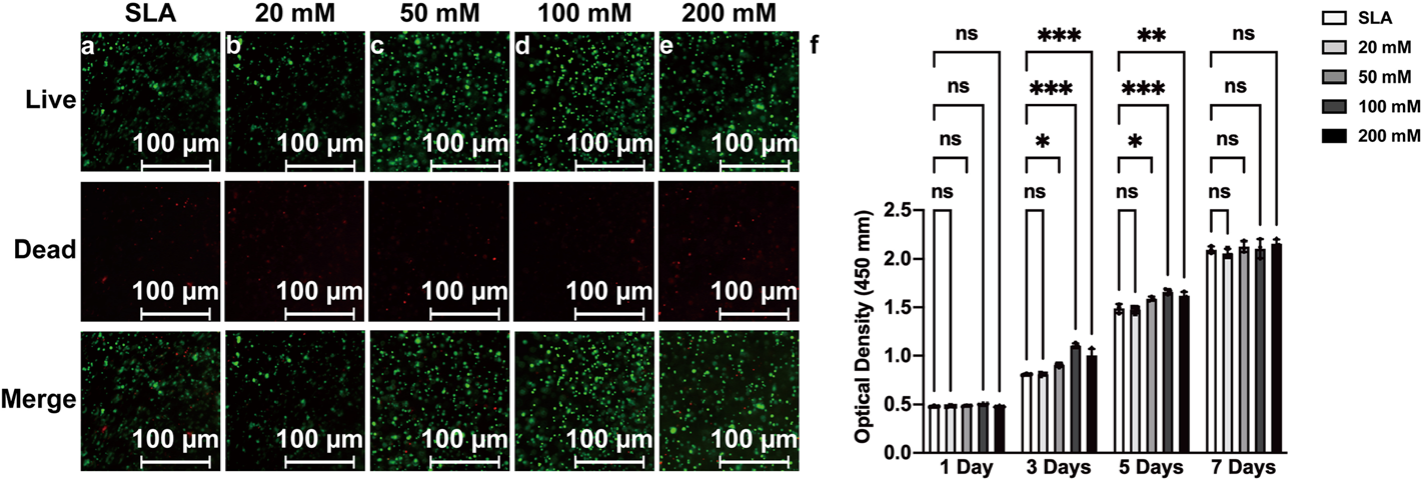
Fluorescence microscopy images of live/dead staining of BMSCs cultured on SLA surfaces (**a**) and SLA discs treated with 20 mM (**b**), 50 mM (**c**), 100 mM (**d**), or 200 mM (**e**) sodium bicarbonate after 72 h of cell seeding. Proliferation of BMSCs cultured in each group for 1, 3, 5, and 7 days determined by CCK-8 assays (**f**). Data are presented as the mean ± standard deviation (*n* = 3, *P < 0.05; **P < 0.01; ***P < 0.001)

Live and dead cell staining was then carried out to investigate the effect of different SLA surfaces on BMSC viability. The results showed that there was no significant difference between SLA discs and SLA discs treated with different concentrations of sodium bicarbonate and further indicated that all the groups had good cytocompatibility (Fig. 7a–e).

The proliferation of BMSCs was assessed by CCK-8 assays, and the results showed that SLA discs treated with 50 mM, 100 mM, and 200 mM sodium bicarbonate significantly enhanced cell proliferation compared with that of the other groups at days 3 and 5 after cell seeding, which may be due to early adhesion and spreading. There was no significant difference between each group after 7 days (Fig. 7f).

### Osteogenic activity of BMSCs

**Fig. 8:**
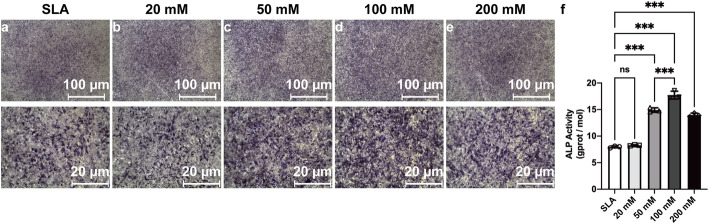
ALP staining images of BMSCs cultured on SLA surfaces (**a**) and SLA discs treated with 20 mM (**b**), 50 mM (**c**), 100 mM (**d**), or 200 mM (**e**) sodium bicarbonate after cell osteogenic differentiation for 7 days. Quantitative results of the relative activity of ALP in BMSCs on different surfaces after 7 days (**f**). Data are presented as the mean ± standard deviation (*n* = 3, *P < 0.05; **P < 0.01; ***P < 0.001)

**Fig. 9:**
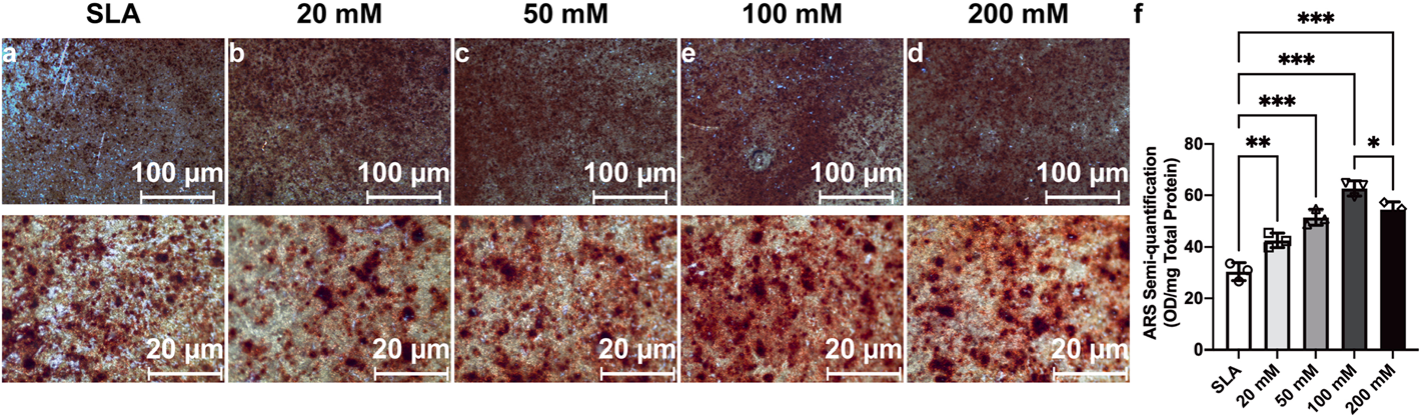
Alizarin Red S (ARS) staining images of calcium deposition of BMSCs cultured on SLA surfaces (**a**) and SLA discs treated with 20 mM (**b**), 50 mM (**c**), 100 mM (**d**), 200 mM (**e**) sodium bicarbonate after cell osteogenic differentiation for 21 days. Semiquantitative results of ARS staining of BMSCs on different surfaces for 21 days (**f**). Data are presented as the mean ± standard deviation (*n* = 3, *P < 0.05; **P < 0.01; ***P < 0.001)

After 7 days of differentiation on the sample discs, BMSCs grown on the discs treated with 100 mM sodium bicarbonate exhibited stronger ALP staining than those treated with other concentrations of sodium bicarbonate as well as the SLA discs (Fig. 8a–e). Likewise, higher ALP activity per cell unit was found for the discs treated with 100 mM sodium bicarbonate (Fig. 8f). Increased calcium deposition, stained with Alizarin Red S, was detected in the discs treated with 100 mM sodium bicarbonate after 21 days of osteogenesis (Fig. 9a–e). Semiquantitative analysis further confirmed this trend (Fig. 9f).

### Gene expression related to BMSC adhesion and differentiation determined by quantitative real-time PCR

**Fig. 10:**
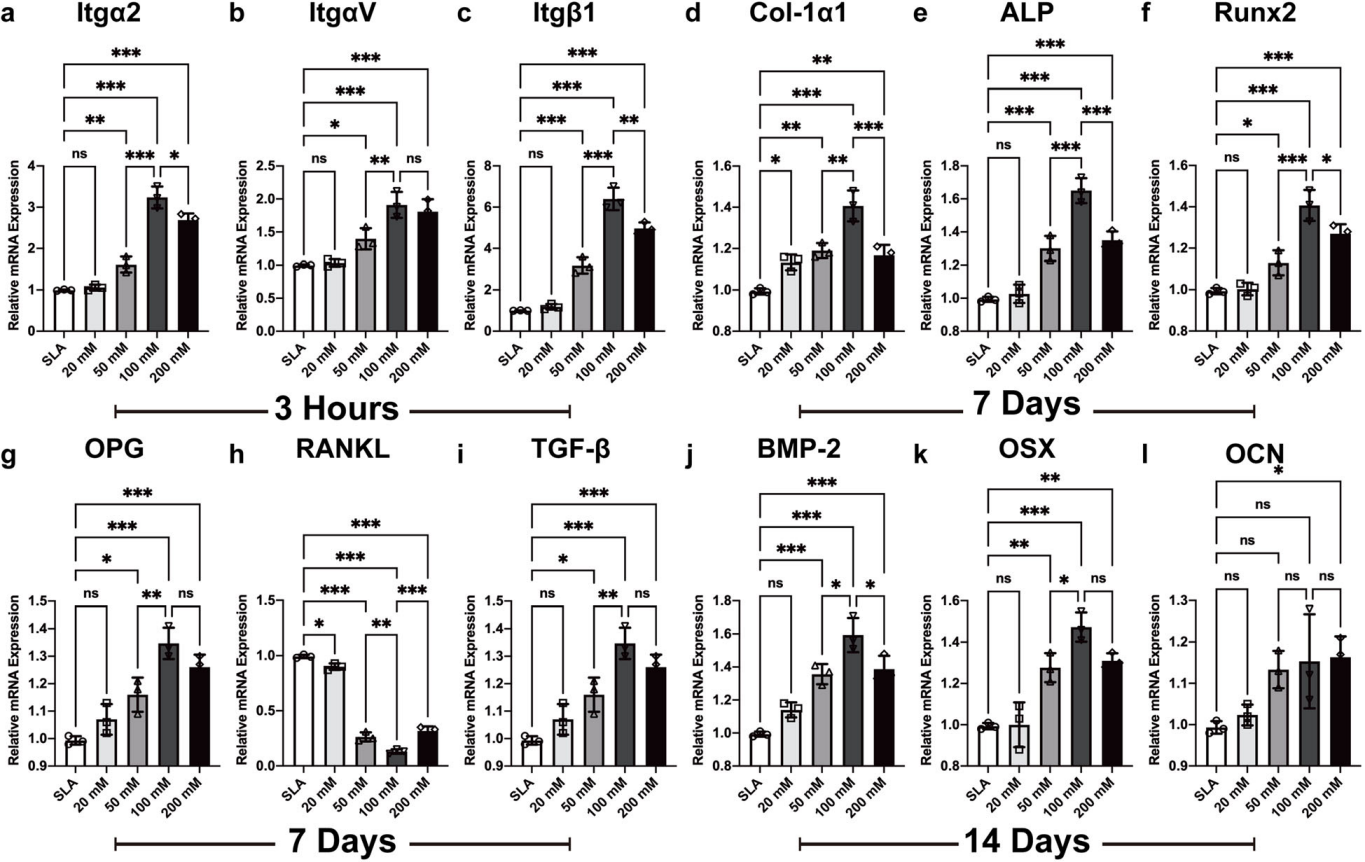
The mRNA expression of integrin α2 (**a**), αV (**b**), and β1 (**c**) at 3 h; Col-1α1 (**d**), Runx2 (**e**), ALP (**f**), OPG (**g**), RANKL (**h**), and aTGF-β (**i**) t 7 days; and BMP-2 (**j**), OSX (**k**), and OCN (**l**) at 14 days after BMSC seeding and osteogenic differentiation on SLA discs treated with different concentration of sodium bicarbonate. Data are presented as the mean ± standard deviation (*n* = 3, *P < 0.05; **P < 0.01; ***P < 0.001)

The cells were seeded on discs treated with different concentrations of sodium bicarbonate. After 3 h of incubation, the mRNA expression levels of integrin a2 (Itg a2), integrin av (Itg av), and integrin β1 (Itg β1) were quantified by qRT-PCR. Compared with the other discs, the discs treated with 100 mM sodium bicarbonate exhibited the most significant increases in integrin α2, integrin αV, and integrin β1 expression (Fig. 10a–c).

To further investigate the expression levels of osteogenesis-related genes in each group, we performed qRT-PCR after 7 and 14 days of osteogenic differentiation of BMSCs. In general, the cells cultured on the discs treated with 100 mM sodium bicarbonate showed higher levels of gene expression than the cells in the other groups. The expression of type 1 collagen (Col-1α), alkaline phosphatase (ALP), runt-related transcription factor 2 (RUNX2), bone sialoprotein (BSP), and transforming growth factor-β (TGF-β) and the ratio of osteoprotegerin to receptor activator of nuclear factor-κ B ligand (OPG/RANKL) at day 7 and osterix (OSX), osteocalcin (OCN), and bone morphogenetic protein 2 (BMP-2) expression at day 14 were considerably higher than those in the control group (Fig. 10d–l).

### Protein expression of BMSC adhesion and differentiation shown by Western blots

**Fig. 11:**
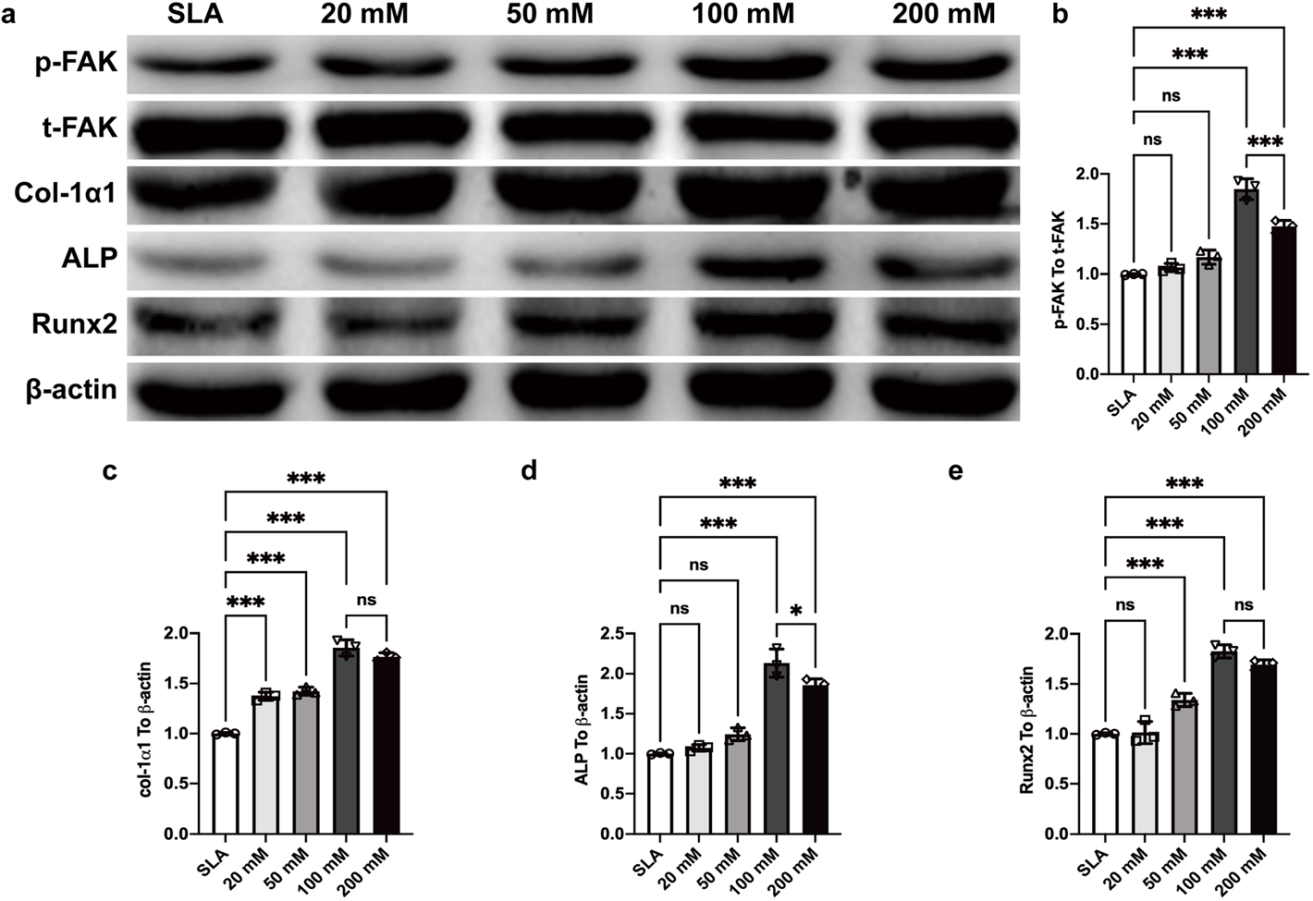
Immunoblot analysis of the expression of phosphorylated and total FAK at day 3 and col-1α1, ALP, Runx2 and β-actin at day 7 after BMSC seeding and osteogenic differentiation on SLA discs treated with different concentrations of sodium bicarbonate (**a**). Semiquantitative analysis of immunoblots of phosphorylation of total FAK (**b**), col-1α1 (**c**), ALP (**d**) and Runx2 (**e**) normalized to β-actin by ImageJ. Data are presented as the mean ± standard deviation (*n* = 3, *P < 0.05; **P < 0.01; ***P < 0.001)

The mRNA expression level of integrin receptors was upregulated in the SLA discs treated with sodium bicarbonate. These receptors can bind to collagen and fibronectin in the extracellular matrix (ECM) and thus activate focal adhesion kinase (FAK) for signaling pathway transmission. We then detected the phosphorylation and total FAK protein expression by Western blots (WBs), and the results showed that 100 mM sodium bicarbonate significantly increased the phosphorylation of FAK at day 3 (Fig. 11a, b). The protein expression of col-1α1, ALP, and Runx2 was upregulated in the SLA discs treated with 100 mM sodium bicarbonate at day 7, further confirming that the early adhesion and proliferation of BMSCs led to osteogenic differentiation (Fig. 11c–e).

## Discussion

In this study, we aimed to evaluate whether the treatment of SLA titanium discs with different concentrations of sodium bicarbonate would affect their biological activity and further regulate BMSC osteogenic differentiation. The results showed that 100 mM sodium bicarbonate could promote BMSC osteogenic differentiation. Due to its high biocompatibility and low cost, sodium bicarbonate has the potential to be applied in dentistry as a solution to conserve dental implants.

To date, most titanium implants have been stored under ambient conditions. Theoretically, titanium or the titanium oxide surface layer, which is produced on titanium contact with atmospheric oxygen, is a high-energy surface [8]. However, the high surface energy enhances adsorption of hydrocarbons from the atmosphere and further decreases the surface energy. Given that this conversion occurs in a time-dependent way, this behavior has been defined as biological aging [4, 22, 23]. Several previous studies investigated the biological aging of the titanium surface, which could impact the osteoconductive and osteoinductive abilities of implants [7, 24, 25]. One important change in aging SLA titanium implants is the conversion of hydrophilic surfaces to hydrophobic surfaces due to carbon contamination [26, 27]. Our results showed soaking in sodium bicarbonate with a concentration above 20 mM significantly enhanced the hydrophilicity of titanium surfaces. Tugulu et al. believed this change could be explained by the cavities on SLA titanium surfaces, which filled with liquid after immersion into any solution due to capillary forces [28]. We also found that increased concentration of sodium bicarbonate improved the surface hydrophilicity, which was probably due to changes in the concentration of hydroxyl groups [29]. Moreover, our previous studies have shown that the effect of sodium bicarbonate immersion on titanium discs was reduced by extensive rinsing [18, 19]. In this study, we immersed the discs into sodium bicarbonate without rinsing shortly before BM seeding to simulate sodium bicarbonate preservation of dental implants. The results showed this experimental procedure had a predictable effect on the pH of cell culture media (Fig. 4b). The reproducibility of the results might come from the stable surface hydrophilicity and elemental components after sodium bicarbonate immersion, which limit the sodium bicarbonate affinity to titanium [18]. Thus, this method may prevent the aging of titanium surfaces, and a suitable concentration of sodium bicarbonate for implant storage is above 20 mM.

In addition to these findings, once the concentration of sodium bicarbonate was within the range of 0–200 mM, it could obviously affect the pH value of the solution at room temperature. When the concentration was 100 mM, the pH value reached a maximum, approximately 9 (Fig. 4). Interestingly, we found that this variation further affected the pH value of cell culture media. Although this study did not clearly identify this phenomenon, our results suggested a correlation between the increased pH of the initial media after BMSC seeding and the pH of different concentrations of sodium bicarbonate solution for titanium disc immersion. Previous research demonstrated that the extracellular pH value is one of the significant aspects with respect to cells in contact with titanium surfaces. The extracellular acid-base equilibrium influences the function of bone cells and bone mineralization processes. There is a rapid decrease in the pH value of the surrounding areas of implants at the early phase of the bone healing process, which is due to the tissue inflammatory response and osteoclast activity [30, 31]. Therefore, a higher extracellular pH value is thought to be beneficial for enhancing osteoblast differentiation and inhibiting osteoclast differentiation [32, 33]. Liu et al. reported a biodegradable implant made of β-tricalcium phosphate (β-TCP), calcium silicate (CS), and 10% strontium-substituted calcium silicate (Sr-CS) powders by a chemical precipitation method, which could enhance the osteoblast differentiation and accelerate the repair of osteoporotic bone defects [31]. Li et al. designed a Mg-Fe layered double hydroxide (LDH) film coating on commercial titanium surfaces via hydrothermal treatment, and by increasing microenvironment pH value, this new method showed good biocompatibility and osteogenic activity both in vitro and in vivo [34]. Our results suggest that titanium discs soaked in sodium bicarbonate successfully promoted the pH value of cell culture media for 5–7 days (Fig. 4b) and further enhanced BMSC adhesion, proliferation, and differentiation (Figs. 5, 6, 7, 8, 9). This finding may be due to the activation of integrin receptors in this weakly alkaline environment, which stimulated the phosphorylation of FAK and further led to the upregulation of ALP and Runx2 expression (Figs. 10–11). Therefore, a suitable and continuous alkaline environment would promote early osteointegration between bone and titanium surfaces. With the rapid development of coating technology, several new strategies have been developed for modification of pure titanium surfaces, such as nanotubes, nanoparticles, and nanofibers [35]. Interestingly, these structures have the ability to conserve small molecules, which made this facile sodium bicarbonate soaking design even more promising in implant conservation.

Several limitations of this study must be considered when interpreting the above data. The pH of cell culture media mentioned above cannot be precisely ascertained, because we did not find a feasible probe to detect the interaction between the cell membrane and the material surfaces. The current results could partially reflect the effect of sodium bicarbonate immersion, which is also affected by factors such as cell metabolism. Additionally, in vitro findings do not always translate to in vivo results. Here, we measured the variation in the extracellular pH value to evaluate the BMSC osteogenic ability, and therefore, we did not account for the competition of other cell lines or the effects of tissue fluid and blood pH balance, which may be affected by ventilation and respiration of the body. However, implants are always placed in a local area, and soaking in sodium bicarbonate could cause a cellular scale change, which could be recognized as a generalized gradient. This local gradient is expected to generate a broader tissue response, and further experiments are needed. In addition, in regard to the potential solutions for storing implants, an in vitro study is needed.

## Conclusion

The present results showed that titanium discs treated with sodium bicarbonate at concentrations of 20–100 mM increased sodium bicarbonate in culture media and thus promoted BMSC osteogenesis in a dose-dependent manner. Implants immersed in 100 mM sodium bicarbonate showed the greatest potential to improve BMSC performance via the ITG-FAK-ALP signaling pathway, and thus, our results suggest a novel conservation method for dental implants.

## Data Availability

All data generated or analyzed during this study are included in this published article.

## Abbreviations

BMSCs: Bone mesenchymal stem cells
SLA: Sand-blasting and acid etching
Itg: Integrin
FAK: Focal adhesion kinase
UV: Ultraviolet
NTAPPJ: Nonthermal atmospheric pressure plasma
ALN: Alendronate
pH: Power of hydrogen ions
SEM: Scanning electron microscope
*R*_a_: Roughness average
*S*_a_: Surface roughness
α-MEM: α-Minimal essential media
FBS: Fetal bovine serum
BCA: Bicinchoninic acid
ALP: Alkaline phosphatase
qRT-PCR: Quantitative real-time PCR
WB: Western blot
RUNX2: Runt-related transcription factor 2
BSP: Bone sialoprotein
TGF-β: Transforming growth factor-β
OPG: Osteoprotegerin
RANKL: Receptor activator of nuclear factor-κ B ligand
OSX: Osterix
OCN: Osteocalcin
BMP-2: Bone morphogenetic protein 2
GAPDH: Glyceraldehyde 3-phosphate dehydrogenase
ARS: Alizarin Red S
CCK8: Cell counting kit-8
ECM: Extracellular matrix
